# Supplementary material for: The Evolution and Characterization of the RNA Interference Pathways in Lophotrochozoa
Source: Genome Biol Evol. 2024 May 7;16(5):evae098. doi: 10.1093/gbe/evae098 (PMC11114477; doi:10.1093/gbe/evae098)
Supplement: evae098_Supplementary_Data [file evae098_supplementary_data.zip › Supplementary_materials.zip!/selected_miRNAs/NoGe_selected_miRNAs/mirdeep_pdf_files/NMRB01000795.1_Notospermus_geniculatus_strain_Ushimado_scaffold795,_whole_genome_shotgun_sequence_20945.pdf]

Provisional ID	:	NMRB01000795.1_Notospermus_geniculatus_strain_Ushimado_scaffold795,_whole_genome_shotgun_sequence_20945
Score total	:	179881.7
Score for star read(s)	:	3.9
Score for read counts	:	179876.6
Score for mfe	:	1.8
Score for randfold	:	0
Score for cons. seed	:	-0.6
Total read count	:	352832
Mature read count	:	352660
Loop read count	:	0
Star read count	:	172

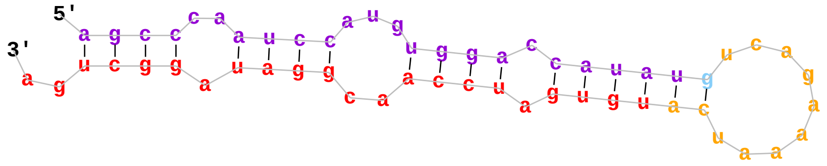

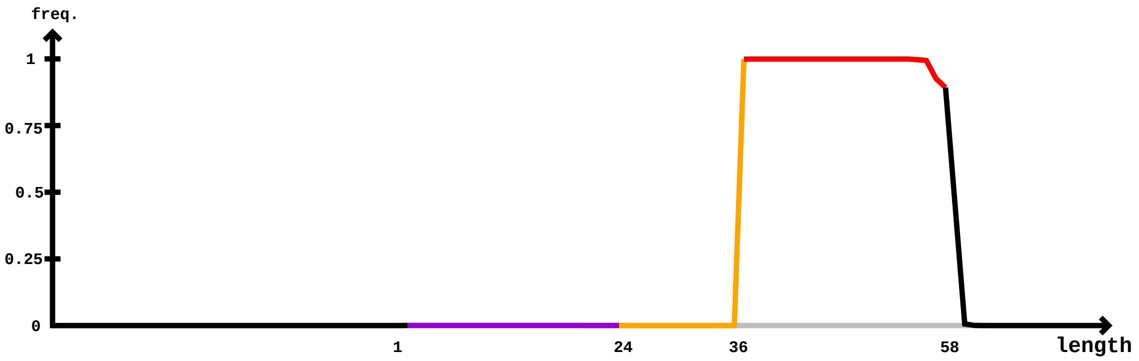


```
                                    Star                                  Mature
5'- ggucauggaaucacgccuagaccuguuuuccuuucagcccaauccauguggaccauaugucagaaaaucaugugauccaacggauaggcugaaagcaaaacaaaaugc   -3'   obs
    ggucauggaaucacgccuagaccuguuuuccuuucagcccaauccauguggaccauaugucagaaaaucaugugauccaacggauaggcugaaagcaaaacaaaaugc         exp
    ((((..((.......))..))))((((((.(((((((((..((((...((((.((((((.........)))))).)))).)..)))).))))))))).))))))......   reads   mm   sample
    ......................................................................ugugauccaacggauagU....................   1       1    Fm3
    ......................................................................ugugauccaacggauagA....................   3       1    Fm3
    ......................................................................ugugauccaacggauGggc...................   1       1    Fm3
    ......................................................................ugugauccaacggauaggU...................   2       1    Fm3
    ......................................................................ugugauccaacggauaggcu..................   17      0    Fm3
    ......................................................................ugugauccaacggauGggcu..................   4       1    Fm3
    ......................................................................ugugauccaacggauaggcuU.................   2       1    Fm3
    ......................................................................ugugauccaacggauaggAug.................   1       1    Fm3
    ......................................................................ugugauccaacggauaggcug.................   7       0    Fm3
    ......................................................................ugugauccaacggauaCgcuga................   1       1    Fm3
    ......................................................................ugugauccaacggauaggcugU................   205     1    Fm3
    ......................................................................Ggugauccaacggauaggcuga................   1       1    Fm3
    ......................................................................Ngugauccaacggauaggcuga................   1       1    Fm3
    ......................................................................ugugauccaacggauagCcuga................   3       1    Fm3
    ......................................................................ugugauccaaUggauaggcuga................   1       1    Fm3
    ......................................................................ugugauccaacggauaggAuga................   1       1    Fm3
    ......................................................................ugugauccaacggauaggcuga................   370     0    Fm3
    ......................................................................ugugauUcaacggauaggcuga................   1       1    Fm3
    ......................................................................ugCgauccaacggauaggcuga................   1       1    Fm3
    ......................................................................ugugauccaacggauaUgcuga................   1       1    Fm3
    ......................................................................ugugauccaacggauagUcuga................   1       1    Fm3
    ......................................................................ugugauccaacggauGggcuga................   16      1    Fm3
    ......................................................................ugugauccaacggauaggcugaU...............   7       1    Fm3
    ......................................................................ugugauccaacggauagCcugaa...............   1       1    Fm3
    ......................................................................ugugauccaaUggauaggcugaa...............   1       1    Fm3
    ......................................................................ugugauccaacggauaggcugaa...............   16      0    Fm3

    ...................................aAcccaauccauguggac.......................................................   1       1    Mm3
    ...................................agcccaauccauguggacc......................................................   1       0    Mm3
    ...................................agcccaauccauguggaccau....................................................   3       0    Mm3
    ...................................agcccaauccauguggaccaua...................................................   4       0    Mm3
    ...................................agcccaauccauguggaAcauau..................................................   1       1    Mm3
    ...................................agcccaauccauguggaccauau..................................................   13      0    Mm3
```

Star **Mature**

```
ggucauggaaucacgccuagaccuguuuuccuuucagcccaauccauguggaccauaugucagaaaaucaugugauccaacggauaggcugaaagcaaaacaaaaugc
...................................agcccaauccauguggaccauauU.................................................   4      1   Mm3
...................................agcccaauccauguggaccauaug.................................................   1      0   Mm3
....................................gcccaauccauguggaccau....................................................   1      0   Mm3
....................................gcccaauccauguggaccauau..................................................   1      0   Mm3
...............................................................aaaaucaugugauccaacggau.......................   2      0   Mm3
....................................................................caugugauccaacggauaggcu..................   1      0   Mm3
.....................................................................Cugugauccaacggauaggcuga................   2      1   Mm3
......................................................................ugugauccaacggauagA....................   28     1   Mm3
......................................................................ugugauccaacggauagg....................   10     0   Mm3
......................................................................ugugauccaacggauagC....................   1      1   Mm3
......................................................................ugugauccaacggauagU....................   107    1   Mm3
......................................................................ugugauccaacggauaggU...................   33     1   Mm3
......................................................................ugugauccaacggauaAgc...................   1      1   Mm3
......................................................................ugugauccaacggauGggc...................   1      1   Mm3
......................................................................ugugauccaacggauaggc...................   120    0   Mm3
......................................................................ugugauccaacggauaggA...................   5      1   Mm3
......................................................................ugugauccaacggauagUc...................   1      1   Mm3
......................................................................ugugauccaacggauaUgc...................   1      1   Mm3
......................................................................ugugauccaacgUauaggcu..................   1      1   Mm3
......................................................................ugugauccaacggauCggcu..................   1      1   Mm3
......................................................................ugugauccaaGggauaggcu..................   3      1   Mm3
......................................................................ugugauUcaacggauaggcu..................   1      1   Mm3
......................................................................ugCgauccaacggauaggcu..................   2      1   Mm3
......................................................................uguAauccaacggauaggcu..................   1      1   Mm3
......................................................................ugugauccaacggauGggcu..................   61     1   Mm3
......................................................................ugugauccaacggauUggcu..................   1      1   Mm3
......................................................................ugugauccaGcggauaggcu..................   1      1   Mm3
......................................................................ugugauccaacggauaCgcu..................   9      1   Mm3
......................................................................ugugauccaacggauaggcG..................   200    1   Mm3
......................................................................ugugauccaaUggauaggcu..................   8      1   Mm3
......................................................................ugugauccaaAggauaggcu..................   9      1   Mm3
......................................................................ugugauccaacggaAaggcu..................   1      1   Mm3
......................................................................ugugCuccaacggauaggcu..................   2      1   Mm3
......................................................................ugGgauccaacggauaggcu..................   1      1   Mm3
......................................................................ugugauccaacggauaggUu..................   2      1   Mm3
......................................................................Ngugauccaacggauaggcu..................   3      1   Mm3
......................................................................ugugauccUacggauaggcu..................   1      1   Mm3
......................................................................ugugauccaacggaCaggcu..................   1      1   Mm3
......................................................................ugugauNcaacggauaggcu..................   1      1   Mm3
......................................................................Agugauccaacggauaggcu..................   1      1   Mm3
......................................................................ugugauccaacggauaAgcu..................   10     1   Mm3
......................................................................ugugaCccaacggauaggcu..................   1      1   Mm3
......................................................................ugugauccaacggGuaggcu..................   1      1   Mm3
......................................................................ugugauccaacgAauaggcu..................   1      1   Mm3
......................................................................ugugauAcaacggauaggcu..................   1      1   Mm3
......................................................................ugugauccaacggauaggcA..................   25     1   Mm3
......................................................................ugugauccaacggauagCcu..................   30     1   Mm3
......................................................................ugugauccaacggauagAcu..................   33     1   Mm3
......................................................................ugugauccaacggauaUgcu..................   7      1   Mm3
......................................................................ugugauccaacggauaggcu..................   5231   0   Mm3
......................................................................ugugauccaacggaGaggcu..................   1      1   Mm3
......................................................................ugugauccaacggauaggcC..................   18     1   Mm3
......................................................................ugAgauccaacggauaggcu..................   2      1   Mm3
......................................................................uAugauccaacggauaggcu..................   2      1   Mm3
......................................................................ugugauccaacggauagUcu..................   22     1   Mm3
......................................................................Cgugauccaacggauaggcu..................   2      1   Mm3
......................................................................ugugauccaCcggauaggcu..................   1      1   Mm3
......................................................................ugugauccaacggauaggcuU.................   66     1   Mm3
......................................................................ugugauccaaAggauaggcug.................   3      1   Mm3
......................................................................ugugauccaacggauaggcuA.................   75     1   Mm3
......................................................................uCugauccaacggauaggcug.................   1      1   Mm3
......................................................................ugugauccaacggauaggcug.................   1346   0   Mm3
......................................................................ugugauccaacggauGggcug.................   24     1   Mm3
......................................................................ugGgauccaacggauaggcug.................   2      1   Mm3
......................................................................ugugauccaacggauaCgcug.................   3      1   Mm3
......................................................................ugugauccaacggauaggcGg.................   21     1   Mm3
......................................................................Agugauccaacggauaggcug.................   1      1   Mm3
......................................................................ugugauccaaUggauaggcug.................   1      1   Mm3
......................................................................ugugauccaacggauaAgcug.................   4      1   Mm3
......................................................................ugugauccaacggauaUgcug.................   2      1   Mm3
```

```
                                                   Star                               Mature
ggucauggaaucacgccuagaccuguuuuccuuucagcccaauccauguggaccauaugucagaaaaucaugugauccaacggauaggcugaaagcaaaacaaaaugc
......................................................................ugugauccaaGggauaggcug.................   3       1   Mm3
......................................................................ugugauccaacggaCaggcug.................   1       1   Mm3
......................................................................ugugauccaacggauagUcug.................   10      1   Mm3
......................................................................ugugauccaacggauagAcug.................   7       1   Mm3
......................................................................ugugaNccaacggauaggcug.................   1       1   Mm3
......................................................................ugugGuccaacggauaggcug.................   1       1   Mm3
......................................................................ugugauccaacggauagCcug.................   4       1   Mm3
......................................................................uguCauccaacggauaggcuga................   1       1   Mm3
......................................................................uAugauccaacggauaggcuga................   11      1   Mm3
......................................................................ugugauccaaGggauaggcuga................   19      1   Mm3
......................................................................ugugauccaacggauUggcuga................   4       1   Mm3
......................................................................ugugauccaacggauaggcuCa................   2       1   Mm3
......................................................................ugugauccaacggUuaggcuga................   4       1   Mm3
......................................................................ugugauccaacgCauaggcuga................   2       1   Mm3
......................................................................ugugauccaacggauaggAuga................   1       1   Mm3
......................................................................ugugaNccaacggauaggcuga................   2       1   Mm3
......................................................................ugugauccaacgUauaggcuga................   1       1   Mm3
......................................................................uCugauccaacggauaggcuga................   3       1   Mm3
......................................................................ugugauUcaacggauaggcuga................   1       1   Mm3
......................................................................ugugaucUaacggauaggcuga................   11      1   Mm3
......................................................................ugugauccaacggaCaggcuga................   1       1   Mm3
......................................................................ugugauccaacggauaggUuga................   4       1   Mm3
......................................................................ugugauccaaUggauaggcuga................   18      1   Mm3
......................................................................ugugauccCacggauaggcuga................   1       1   Mm3
......................................................................ugugauccaacAgauaggcuga................   4       1   Mm3
......................................................................ugugauccaGcggauaggcuga................   3       1   Mm3
......................................................................ugugaucGaacggauaggcuga................   1       1   Mm3
......................................................................ugugauccaacggauaUgcuga................   34      1   Mm3
......................................................................ugugauccaCcggauaggcuga................   1       1   Mm3
......................................................................ugugUuccaacggauaggcuga................   2       1   Mm3
......................................................................uguAauccaacggauaggcuga................   2       1   Mm3
......................................................................ugugauccaacggauagAcuga................   105     1   Mm3
......................................................................ugugGuccaacggauaggcuga................   3       1   Mm3
......................................................................ugGgauccaacggauaggcuga................   8       1   Mm3
......................................................................ugugauccGacggauaggcuga................   2       1   Mm3
......................................................................ugugauccaacggauaggcuga................   17641   0   Mm3
......................................................................ugugauccaacUgauaggcuga................   2       1   Mm3
......................................................................ugugauccaacggauaggcugG................   259     1   Mm3
......................................................................ugugaAccaacggauaggcuga................   4       1   Mm3
......................................................................ugugauccUacggauaggcuga................   6       1   Mm3
......................................................................ugugCuccaacggauaggcuga................   2       1   Mm3
......................................................................ugugauccaUcggauaggcuga................   1       1   Mm3
......................................................................ugugauccaacggauagUcuga................   89      1   Mm3
......................................................................ugugauccaacggauaggcugC................   23      1   Mm3
......................................................................ugAgauccaacggauaggcuga................   2       1   Mm3
......................................................................ugugauccaacggauagCcuga................   95      1   Mm3
......................................................................ugugaucAaacggauaggcuga................   1       1   Mm3
......................................................................ugugauAcaacggauaggcuga................   2       1   Mm3
......................................................................uUugauccaacggauaggcuga................   1       1   Mm3
......................................................................ugugauccaacggauGggcuga................   233     1   Mm3
......................................................................ugugauccaacggaAaggcuga................   1       1   Mm3
......................................................................ugugauccaacggauaCgcuga................   46      1   Mm3
......................................................................ugugauccaacggauaggcugU................   6758    1   Mm3
......................................................................Ngugauccaacggauaggcuga................   10      1   Mm3
......................................................................Agugauccaacggauaggcuga................   3       1   Mm3
......................................................................ugugauccaacggCuaggcuga................   3       1   Mm3
......................................................................ugugauccaaAggauaggcuga................   23      1   Mm3
......................................................................ugCgauccaacggauaggcuga................   4       1   Mm3
......................................................................ugugaCccaacggauaggcuga................   1       1   Mm3
......................................................................ugugauccaacCgauaggcuga................   1       1   Mm3
......................................................................ugugauccaacggauaggcuUa................   3       1   Mm3
......................................................................Ggugauccaacggauaggcuga................   1       1   Mm3
......................................................................ugugauccaacggauaggcCga................   2       1   Mm3
......................................................................uguUauccaacggauaggcuga................   3       1   Mm3
......................................................................ugugauccaacggauaAgcuga................   27      1   Mm3
......................................................................ugugauccaacggauaggcuAa................   15      1   Mm3
......................................................................ugugauccaacggauGggcugaa...............   7       1   Mm3
......................................................................ugugauccaacggauagAcugaa...............   4       1   Mm3
......................................................................ugugauccaacggauaggcugaG...............   6       1   Mm3
......................................................................ugugauccaacggauagUcugaa...............   4       1   Mm3
```

```
                                   Star                                Mature
ggucauggaaucacgccuagaccuguuuuccuuucagcccaauccauguggaccauaugucagaaaaucaugugauccaacggauaggcugaaagcaaaacaaaaugc
......................................................................ugugauccaacggauaggUuga................   2     1    Mm3
......................................................................ugugauccaacggauaggcugUa...............   67    1    Mm3
......................................................................ugugaucUaacggauaggcugaa...............   1     1    Mm3
......................................................................ugugauccaacAgauaggcugaa...............   1     1    Mm3
......................................................................ugugauccaaUggauaggcugaa...............   1     1    Mm3
......................................................................ugugauccaacggauaggcugaa...............   1281  0    Mm3
......................................................................ugugauccaacggauagCcugaa...............   3     1    Mm3
......................................................................uAugauccaacggauaggcugaa...............   1     1    Mm3
......................................................................ugugauccaacggauaCgcugaa...............   6     1    Mm3
......................................................................ugugaNccaacggauaggcugaa...............   1     1    Mm3
......................................................................ugugaucGaacggauaggcugaa...............   1     1    Mm3
......................................................................ugugauccaacggauaggcuAaa...............   6     1    Mm3
......................................................................ugugauccaacggauaAgcugaa...............   3     1    Mm3
......................................................................ugugauccaGcggauaggcugaa...............   1     1    Mm3
......................................................................ugugauccaaAggauaggcugaa...............   2     1    Mm3
......................................................................ugugauccaaGggauaggcugaa...............   3     1    Mm3
......................................................................ugugauccaacggauaggcugCa...............   1     1    Mm3
......................................................................ugugauccaacggauaggcugaU...............   480   1    Mm3
......................................................................ugugauccaacggauaggcugaaG..............   6     1    Mm3
......................................................................ugugauccaacggauaggcugUaa..............   10    1    Mm3
......................................................................ugugauccaacggauaggcuAaaa..............   1     1    Mm3
......................................................................ugugauccaacggauaggcugaaC..............   1     1    Mm3
......................................................................ugugauccaacggauaAgcugaaa..............   1     1    Mm3
......................................................................ugugauccaacggauaggcugaaa..............   90    0    Mm3
......................................................................ugugauccaacggauaggcugaGa..............   3     1    Mm3
......................................................................ugugauccaacggauaggcugaaU..............   82    1    Mm3
......................................................................ugugauccaacggauaggcugaUa..............   8     1    Mm3
......................................................................ugugauUcaacggauaggcugaaa..............   1     1    Mm3
......................................................................ugugauccaacggauGggcugaaa..............   3     1    Mm3
......................................................................ugugauccaacggauaggcugaaag.............   1     0    Mm3
......................................................................ugugauccaacggauaggcugaaaU.............   15    1    Mm3
......................................................................ugugauccaacggauaggcugaaaA.............   8     1    Mm3
......................................................................ugugauccaacggauaggcugaaagU............   1     1    Mm3
......................................................................ugugauccaacggauaggcugaaagAaaa.........   1     1    Mm3
.......................................................................gugauccaacggauaggcu..................   2     0    Mm3
.......................................................................gugauccaacggauaggcug.................   2     0    Mm3
.......................................................................gugauccaacUgauaggcug.................   1     1    Mm3
.......................................................................Uugauccaacggauaggcuga................   1     1    Mm3
.......................................................................gugauccaacggauaggcuga................   5     0    Mm3
.......................................................................gugauccaacggauGggcuga................   1     1    Mm3
........................................................................ugauccaacggauaggcug.................   1     0    Mm3
........................................................................ugauccaacggauaggcuga................   2     0    Mm3
........................................................................ugauccaacggauaggcugU................   1     1    Mm3
.........................................................................gauccaacggauaggcuA.................   1     1    Mm3
.........................................................................gauccaacggauaggcug.................   1     0    Mm3
..........................................................................auccaacggaAaggcuga................   1     1    Mm3
..........................................................................auccaacggauaggcuga................   1     0    Mm3

......................................................................ugugauccaacggauaggU...................   1     1    Fm1
......................................................................ugugauccaacggauGggcu..................   1     1    Fm1
......................................................................ugugauccaacggauaggcu..................   7     0    Fm1
......................................................................ugugauccaacggauaAgcug.................   1     1    Fm1
......................................................................ugugauccaacCgauaggcug.................   1     1    Fm1
......................................................................ugugauccaacggauaggcug.................   10    0    Fm1
......................................................................ugugauccaacggauaggcugU................   26    1    Fm1
......................................................................ugAgauccaacggauaggcuga................   1     1    Fm1
......................................................................ugugauccaacggauagAcuga................   1     1    Fm1
......................................................................ugugauccaacggauaggcugC................   8     1    Fm1
......................................................................ugugauccaacggauagUcuga................   1     1    Fm1
......................................................................ugugauccaaAggauaggcuga................   1     1    Fm1
......................................................................ugugauccaacggauaggcuga................   108   0    Fm1
......................................................................ugugauccaacggauGggcuga................   14    1    Fm1
......................................................................ugugauccaacggauaggcugUa...............   1     1    Fm1
......................................................................ugugauccaacggauaggcugaa...............   3     0    Fm1
......................................................................ugugauccaacggauaggcugaU...............   2     1    Fm1
......................................................................ugugauccaacggauaggcugaaU..............   2     1    Fm1

......................................................................ugugauccaacggauaggc...................   2     0    Fm2
......................................................................ugugauccaacggauaggcu..................   9     0    Fm2
......................................................................ugugauccaacggauaggcug.................   6     0    Fm2
```

Star **Mature**

```
ggucauggaaucacgccuagaccuguuuuccuuucagcccaauccauguggaccauaugucagaaaaucaugugauccaacggauaggcugaaagcaaaacaaaaugc
......................................................................ugugauccaacggauaggcuga................  72   0  Fm2
......................................................................ugugauccaacggauGggcuga................  32   1  Fm2
......................................................................ugugauccaacggauagCcuga................  1    1  Fm2
......................................................................ugugauccaacggauaggcugU................  34   1  Fm2
......................................................................ugugauccaacggauaggcugG................  2    1  Fm2
......................................................................Agugauccaacggauaggcuga................  1    1  Fm2
......................................................................ugugauccaacggauaggcugaU...............  4    1  Fm2
......................................................................ugugauccaacggauaggcugaa...............  9    0  Fm2

................cuagaccuguuuuccuuuc.........................................................................  1    0  Mm2
...............................uuucagcccaauccauguggacc......................................................  1    0  Mm2
...................................agcccaauccauguggac.......................................................  1    0  Mm2
...................................agcccaauccauguggaccau....................................................  3    0  Mm2
...................................agcccaauccauguggaccaua...................................................  26   0  Mm2
...................................agcccaauccauguggaccauU...................................................  2    1  Mm2
...................................agcccaauccauguggaccauau..................................................  53   0  Mm2
...................................agcccaauccaCguggaccauau..................................................  1    1  Mm2
...................................agcccaauccauguggaccauaA..................................................  1    1  Mm2
...................................agcccaauccauguggaccauaug.................................................  16   0  Mm2
...................................agcccaauccauguggaccauauU.................................................  4    1  Mm2
...................................agcccaauccauguggaccauaugucaga............................................  4    0  Mm2
.....................................cccaauccauguggaccauaug.................................................  2    0  Mm2
...........................................................ucagaaaaucaugugauccaacggauagg....................  1    0  Mm2
...........................................................ucagaaaaucaugugauAcaacggauagg....................  1    1  Mm2
.................................................................aaucaugugauccaacggauaggU...................  3    1  Mm2
.................................................................aaucaugugauccaacggauaggcu..................  1    0  Mm2
.................................................................aaucaugugauccaacggauaggcuga................  3    0  Mm2
...................................................................ucaugugauccaacggauaggcuga................  1    0  Mm2
....................................................................caugugauccaacggauaggcu..................  6    0  Mm2
.....................................................................Cugugauccaacggauaggcu..................  1    1  Mm2
.....................................................................augugauccaacggauGggcu..................  1    1  Mm2
.....................................................................augugauccaacggauaggcugU................  1    1  Mm2
.....................................................................Cugugauccaacggauaggcuga................  7    1  Mm2
.....................................................................augugauccaacggauaggcuga................  7    0  Mm2
......................................................................ugugauccaacggauaUg....................  1    1  Mm2
......................................................................ugugauccaacggauGgg....................  4    1  Mm2
......................................................................ugugauccaacggauagA....................  35   1  Mm2
......................................................................ugugauccaacggauagg....................  34   0  Mm2
......................................................................ugugauccaacggauagU....................  270  1  Mm2
......................................................................ugugauccaacggauaUgc...................  1    1  Mm2
......................................................................ugCgauccaacggauaggc...................  1    1  Mm2
......................................................................ugugauccaacggauaggc...................  440  0  Mm2
......................................................................ugugauccaacggaCaggc...................  1    1  Mm2
......................................................................ugugauccaacggauaAgc...................  1    1  Mm2
......................................................................ugugauccaacggauagAc...................  2    1  Mm2
......................................................................ugugauccaacggauagCc...................  1    1  Mm2
......................................................................ugugauccaacggauagUc...................  6    1  Mm2
......................................................................ugugauccaacggauaggU...................  55   1  Mm2
......................................................................ugugauccaacggauaggA...................  9    1  Mm2
......................................................................ugugauccaacggauGggc...................  17   1  Mm2
......................................................................ugugauccaacggauaCgc...................  1    1  Mm2
......................................................................ugugauccaacggauagCcu..................  59   1  Mm2
......................................................................ugAgauccaacggauaggcu..................  1    1  Mm2
......................................................................ugugauAcaacggauaggcu..................  1    1  Mm2
......................................................................uguUauccaacggauaggcu..................  2    1  Mm2
......................................................................uCugauccaacggauaggcu..................  2    1  Mm2
......................................................................ugugauGcaacggauaggcu..................  1    1  Mm2
......................................................................ugugauccaacAgauaggcu..................  3    1  Mm2
......................................................................ugugaucGaacggauaggcu..................  1    1  Mm2
......................................................................ugugauccaacggCuaggcu..................  1    1  Mm2
......................................................................ugugaucUaacggauaggcu..................  2    1  Mm2
......................................................................Cgugauccaacggauaggcu..................  2    1  Mm2
......................................................................ugugauccaacggUuaggcu..................  1    1  Mm2
......................................................................ugugauccaacggauagAcu..................  82   1  Mm2
......................................................................ugugaCccaacggauaggcu..................  2    1  Mm2
......................................................................ugugCuccaacggauaggcu..................  1    1  Mm2
......................................................................ugugaAccaacggauaggcu..................  2    1  Mm2
......................................................................ugugauccCacggauaggcu..................  2    1  Mm2
......................................................................ugugaNccaacggauaggcu..................  2    1  Mm2
......................................................................uAugauccaacggauaggcu..................  6    1  Mm2
```

```
                                   Star                               Mature
ggucauggaaucacgccuagaccuguuuuccuuucagcccaauccauguggaccauaugucagaaaaucaugugauccaacggauaggcugaaagcaaaacaaaaugc
......................................................................ugugauccaaUggauaggcu..................  15     1   Mm2
......................................................................ugugauccaacggauGggcu..................  449    1   Mm2
......................................................................ugugauccaacggauaggcu..................  12137  0   Mm2
......................................................................ugugauccGacggauaggcu..................  2      1   Mm2
......................................................................ugugauccaacggaGaggcu..................  1      1   Mm2
......................................................................ugGgauccaacggauaggcu..................  1      1   Mm2
......................................................................ugugauccaCcggauaggcu..................  1      1   Mm2
......................................................................ugugauccaaAggauaggcu..................  18     1   Mm2
......................................................................ugugauccUacggauaggcu..................  10     1   Mm2
......................................................................Agugauccaacggauaggcu..................  1      1   Mm2
......................................................................Ngugauccaacggauaggcu..................  9      1   Mm2
......................................................................uUugauccaacggauaggcu..................  2      1   Mm2
......................................................................ugugauccaacggaAaggcu..................  2      1   Mm2
......................................................................uguCauccaacggauaggcu..................  1      1   Mm2
......................................................................ugugauccaacggauagUcu..................  48     1   Mm2
......................................................................ugugauccaacggauaggcA..................  32     1   Mm2
......................................................................ugugauccaacggauaggcC..................  16     1   Mm2
......................................................................ugugauccaacgAauaggcu..................  1      1   Mm2
......................................................................uguAauccaacggauaggcu..................  2      1   Mm2
......................................................................ugugauccaaGggauaggcu..................  18     1   Mm2
......................................................................ugCgauccaacggauaggcu..................  2      1   Mm2
......................................................................ugugauccaacggauaggcG..................  641    1   Mm2
......................................................................ugugauccaacggauaCgcu..................  27     1   Mm2
......................................................................ugugauccaacggauaAgcu..................  27     1   Mm2
......................................................................ugugauccaacggaCaggcu..................  1      1   Mm2
......................................................................ugugauccaacgUauaggcu..................  1      1   Mm2
......................................................................ugugauccaacggauaUgcu..................  17     1   Mm2
......................................................................ugugauccaacggauUggcu..................  2      1   Mm2
......................................................................ugugauccaacggaGaggcug.................  1      1   Mm2
......................................................................ugugGuccaacggauaggcug.................  2      1   Mm2
......................................................................ugGgauccaacggauaggcug.................  2      1   Mm2
......................................................................ugCgauccaacggauaggcug.................  1      1   Mm2
......................................................................ugugauccaacggauaggcug.................  6493   0   Mm2
......................................................................ugugauccaacggauaUgcug.................  11     1   Mm2
......................................................................ugugauccGacggauaggcug.................  1      1   Mm2
......................................................................ugugauccaacggaCaggcug.................  1      1   Mm2
......................................................................ugugauccaacggauagCcug.................  24     1   Mm2
......................................................................ugugauccaacggUuaggcug.................  1      1   Mm2
......................................................................ugugauccaacggauaggcuA.................  296    1   Mm2
......................................................................ugugaucGaacggauaggcug.................  1      1   Mm2
......................................................................ugugauccaacggaAaggcug.................  1      1   Mm2
......................................................................ugugauccCacggauaggcug.................  2      1   Mm2
......................................................................uUugauccaacggauaggcug.................  4      1   Mm2
......................................................................ugugauccaacggauUggcug.................  1      1   Mm2
......................................................................Cgugauccaacggauaggcug.................  3      1   Mm2
......................................................................Agugauccaacggauaggcug.................  5      1   Mm2
......................................................................ugugauccaacUgauaggcug.................  1      1   Mm2
......................................................................ugAgauccaacggauaggcug.................  3      1   Mm2
......................................................................ugugauccaaUggauaggcug.................  5      1   Mm2
......................................................................ugugauccaacggauaggcuU.................  298    1   Mm2
......................................................................ugugauccaacggauagUcug.................  29     1   Mm2
......................................................................ugugauccaacggauaCgcug.................  16     1   Mm2
......................................................................uCugauccaacggauaggcug.................  2      1   Mm2
......................................................................ugugauccUacggauaggcug.................  3      1   Mm2
......................................................................ugugauccaaGggauaggcug.................  14     1   Mm2
......................................................................ugugauccaacAgauaggcug.................  2      1   Mm2
......................................................................ugugCuccaacggauaggcug.................  1      1   Mm2
......................................................................ugugaAccaacggauaggcug.................  1      1   Mm2
......................................................................uAugauccaacggauaggcug.................  6      1   Mm2
......................................................................ugugauGcaacggauaggcug.................  1      1   Mm2
......................................................................ugugauccaacgUauaggcug.................  1      1   Mm2
......................................................................ugugauccaacggauGggcug.................  165    1   Mm2
......................................................................ugugauccaacggauaggcGg.................  187    1   Mm2
......................................................................Ngugauccaacggauaggcug.................  6      1   Mm2
......................................................................ugugauccaacgCauaggcug.................  1      1   Mm2
......................................................................ugugaucUaacggauaggcug.................  3      1   Mm2
......................................................................ugugauUcaacggauaggcug.................  1      1   Mm2
......................................................................ugugauccaacggauaggAug.................  2      1   Mm2
......................................................................ugugauccaacggauaggcCg.................  4      1   Mm2
......................................................................ugugauccaacggauaAgcug.................  16     1   Mm2
```

Star **Mature**

```
ggucauggaaucacgccuagaccuguuuuccuuucagcccaauccauguggaccauaugucagaaaaucaugugauccaacggauaggcugaaagcaaaacaaaaugc
......................................................................ugugauccaacggGuaggcug.................   1       1   Mm2
......................................................................ugugauccaaAggauaggcug.................   9       1   Mm2
......................................................................ugugauccaacggauaggcuC.................   2       1   Mm2
......................................................................ugugauccaacggauagAcug.................   47      1   Mm2
......................................................................ugugauccaUcggauaggcug.................   1       1   Mm2
......................................................................ugugauccaacggauaggcAga................   6       1   Mm2
......................................................................ugugaAccaacggauaggcuga................   18      1   Mm2
......................................................................ugugauccaUcggauaggcuga................   25      1   Mm2
......................................................................Agugauccaacggauaggcuga................   22      1   Mm2
......................................................................ugugauccaaUggauaggcuga................   181     1   Mm2
......................................................................ugugauccaacggauaggcugC................   137     1   Mm2
......................................................................ugugauccaacggUuaggcuga................   12      1   Mm2
......................................................................ugugauccaaAggauaggcuga................   287     1   Mm2
......................................................................ugugaCccaacggauaggcuga................   15      1   Mm2
......................................................................ugugauccaacggauaggcuAa................   67      1   Mm2
......................................................................ugugauccaacggauaggcuga................   163086  0   Mm2
......................................................................ugugauccaacgAauaggcuga................   6       1   Mm2
......................................................................ugugauccaacggauaggGuga................   6       1   Mm2
......................................................................uCugauccaacggauaggcuga................   16      1   Mm2
......................................................................ugugauccUacggauaggcuga................   77      1   Mm2
......................................................................uguAauccaacggauaggcuga................   28      1   Mm2
......................................................................ugAgauccaacggauaggcuga................   40      1   Mm2
......................................................................ugugauccaacggauaggUuga................   21      1   Mm2
......................................................................ugugauccaacggauUggcuga................   16      1   Mm2
......................................................................ugugauccaacggauaggcCga................   13      1   Mm2
......................................................................ugugauccaacgUauaggcuga................   27      1   Mm2
......................................................................ugugauccaacggCuaggcuga................   9       1   Mm2
......................................................................uUugauccaacggauaggcuga................   25      1   Mm2
......................................................................ugugauccaacggGuaggcuga................   15      1   Mm2
......................................................................ugugauccaacggauCggcuga................   11      1   Mm2
......................................................................ugugauccaacggaAaggcuga................   8       1   Mm2
......................................................................ugugauccaCcggauaggcuga................   15      1   Mm2
......................................................................uAugauccaacggauaggcuga................   71      1   Mm2
......................................................................ugugauNcaacggauaggcuga................   12      1   Mm2
......................................................................ugugauccGacggauaggcuga................   45      1   Mm2
......................................................................uguUauccaacggauaggcuga................   12      1   Mm2
......................................................................Ggugauccaacggauaggcuga................   5       1   Mm2
......................................................................ugugauccCacggauaggcuga................   15      1   Mm2
......................................................................ugugauUcaacggauaggcuga................   26      1   Mm2
......................................................................ugugCuccaacggauaggcuga................   22      1   Mm2
......................................................................ugugauccaacggauaggcugU................   48479   1   Mm2
......................................................................ugugauccaacggauaggAuga................   12      1   Mm2
......................................................................ugugauccaacggauaAgcuga................   309     1   Mm2
......................................................................ugugaGccaacggauaggcuga................   7       1   Mm2
......................................................................ugugaucUaacggauaggcuga................   78      1   Mm2
......................................................................Cgugauccaacggauaggcuga................   19      1   Mm2
......................................................................ugCgauccaacggauaggcuga................   30      1   Mm2
......................................................................ugugauccaacCgauaggcuga................   7       1   Mm2
......................................................................ugugauccaacgCauaggcuga................   4       1   Mm2
......................................................................ugugauccaacggauaggcGga................   4       1   Mm2
......................................................................ugugGuccaacggauaggcuga................   45      1   Mm2
......................................................................ugugauccaacggauaCgcuga................   428     1   Mm2
......................................................................ugugauccaacggauaggcugG................   3288    1   Mm2
......................................................................ugugauGcaacggauaggcuga................   2       1   Mm2
......................................................................uNugauccaacggauaggcuga................   5       1   Mm2
......................................................................ugugauccaacggauGggcuga................   3220    1   Mm2
......................................................................ugugaucGaacggauaggcuga................   2       1   Mm2
......................................................................ugugauccaacggauagCcuga................   757     1   Mm2
......................................................................ugugauccaacggauaggcuCa................   9       1   Mm2
......................................................................ugugauccaacAgauaggcuga................   36      1   Mm2
......................................................................ugugauccaGcggauaggcuga................   18      1   Mm2
......................................................................Ngugauccaacggauaggcuga................   80      1   Mm2
......................................................................ugugauccaaGggauaggcuga................   171     1   Mm2
......................................................................uguCauccaacggauaggcuga................   8       1   Mm2
......................................................................ugugauccaacggaGaggcuga................   8       1   Mm2
......................................................................ugugaNccaacggauaggcuga................   17      1   Mm2
......................................................................ugugaucAaacggauaggcuga................   18      1   Mm2
......................................................................ugugauccaacggauagAcuga................   930     1   Mm2
......................................................................ugugUuccaacggauaggcuga................   22      1   Mm2
......................................................................ugugauccaacggauaggcuUa................   26      1   Mm2
```

```
                                   Star                             Mature
ggucauggaaucacgccuagaccuguuuuccuuucagcccaauccauguggaccauaugucagaaaaucaugugauccaacggauaggcugaaagcaaaacaaaaugc
......................................................................ugGgauccaacggauaggcuga................  39    1  Mm2
......................................................................ugugauccaacggauaUgcuga................  262   1  Mm2
......................................................................ugugauccaacUgauaggcuga................  27    1  Mm2
......................................................................ugugauccaacggauagUcuga................  823   1  Mm2
......................................................................ugugauAcaacggauaggcuga................  16    1  Mm2
......................................................................ugugauccaacggaCaggcuga................  17    1  Mm2
......................................................................uguCauccaacggauaggcugaa...............  1     1  Mm2
......................................................................ugugauccaacggUuaggcugaa...............  1     1  Mm2
......................................................................uguAauccaacggauaggcugaa...............  1     1  Mm2
......................................................................ugCgauccaacggauaggcugaa...............  2     1  Mm2
......................................................................ugugauccaacggauaAgcugaa...............  8     1  Mm2
......................................................................uCugauccaacggauaggcugaa...............  1     1  Mm2
......................................................................Ngugauccaacggauaggcugaa...............  1     1  Mm2
......................................................................ugugauccaacggauaUgcugaa...............  19    1  Mm2
......................................................................ugugauccaacggauGggcugaa...............  111   1  Mm2
......................................................................ugGgauccaacggauaggcugaa...............  3     1  Mm2
......................................................................ugugauccaacggauaggcugUa...............  229   1  Mm2
......................................................................ugugauccaacggauaggcugaU...............  3311  1  Mm2
......................................................................ugugaNccaacggauaggcugaa...............  1     1  Mm2
......................................................................ugugauccaacggauaCgcugaa...............  28    1  Mm2
......................................................................Cgugauccaacggauaggcugaa...............  2     1  Mm2
......................................................................ugAgauccaacggauaggcugaa...............  3     1  Mm2
......................................................................ugugauccaacggauaggcuUaa...............  1     1  Mm2
......................................................................ugugauccaaAggauaggcugaa...............  10    1  Mm2
......................................................................ugugauccaacggauaggcugaG...............  40    1  Mm2
......................................................................ugugauccaacggauaggcCgaa...............  1     1  Mm2
......................................................................ugugauccaacAgauaggcugaa...............  1     1  Mm2
......................................................................ugugauccaacggaCaggcugaa...............  1     1  Mm2
......................................................................ugugauccaacggauaggcugGa...............  1     1  Mm2
......................................................................ugugauccaacggauaggcugaa...............  5918  0  Mm2
......................................................................ugugaucUaacggauaggcugaa...............  2     1  Mm2
......................................................................ugugGuccaacggauaggcugaa...............  1     1  Mm2
......................................................................ugugauccaacggauagCcugaa...............  24    1  Mm2
......................................................................ugugauccaacggauaggcAgaa...............  1     1  Mm2
......................................................................ugugauccaUcggauaggcugaa...............  1     1  Mm2
......................................................................ugugauccaGcggauaggcugaa...............  2     1  Mm2
......................................................................ugugauccaacggauagUcugaa...............  22    1  Mm2
......................................................................ugugauccaacggauUggcugaa...............  1     1  Mm2
......................................................................ugugauccaacggauaggcugaC...............  10    1  Mm2
......................................................................ugugaCccaacggauaggcugaa...............  1     1  Mm2
......................................................................ugugauccaaGggauaggcugaa...............  9     1  Mm2
......................................................................Agugauccaacggauaggcugaa...............  3     1  Mm2
......................................................................ugugauccaaUggauaggcugaa...............  4     1  Mm2
......................................................................ugugauccUacggauaggcugaa...............  3     1  Mm2
......................................................................uUugauccaacggauaggcugaa...............  1     1  Mm2
......................................................................ugugauccaacggauaggcuAaa...............  8     1  Mm2
......................................................................ugugauccGacggauaggcugaa...............  1     1  Mm2
......................................................................ugugauccaCcggauaggcugaa...............  2     1  Mm2
......................................................................ugugauccaacggauagAcugaa...............  42    1  Mm2
......................................................................uguUauccaacggauaggcugaa...............  2     1  Mm2
......................................................................ugugauccaacggCuaggcugaa...............  2     1  Mm2
......................................................................ugugauccaacggauaggcugUaa..............  21    1  Mm2
......................................................................ugugauccaacggauagAcugaaa..............  4     1  Mm2
......................................................................ugugauccaaGggauaggcugaaa..............  2     1  Mm2
......................................................................ugugauccGacggauaggcugaaa..............  1     1  Mm2
......................................................................ugugauccaacggauagCcugaaa..............  5     1  Mm2
......................................................................ugugauccaacggauaggcugaaG..............  61    1  Mm2
......................................................................ugugauccaacggauaggcugaUa..............  82    1  Mm2
......................................................................ugugauccaacgUauaggcugaaa..............  1     1  Mm2
......................................................................ugugauccaacggauaggcugaaC..............  15    1  Mm2
......................................................................ugugauccaacggauaCgcugaaa..............  2     1  Mm2
......................................................................uguCauccaacggauaggcugaaa..............  1     1  Mm2
......................................................................ugugauccaacggauGggcugaaa..............  15    1  Mm2
......................................................................ugugauccaaUggauaggcugaaa..............  1     1  Mm2
......................................................................Agugauccaacggauaggcugaaa..............  1     1  Mm2
......................................................................ugugauGcaacggauaggcugaaa..............  1     1  Mm2
......................................................................ugGgauccaacggauaggcugaaa..............  1     1  Mm2
......................................................................ugugaucGaacggauaggcugaaa..............  1     1  Mm2
......................................................................ugugauccaacggauaggcugaaa..............  680   0  Mm2
......................................................................ugugauccaacggauaggcugaGa..............  24    1  Mm2
```

```
                                   Star                              Mature
ggucauggaaucacgccuagaccuguuuuccuuucagcccaauccauguggaccauaugucagaaaaucaugugauccaacggauaggcugaaagcaaaacaaaaugc
......................................................................ugugauUcaacggauaggcugaaa..............   1     1    Mm2
......................................................................ugugauccaacggauaggcuUaaa..............   1     1    Mm2
......................................................................ugugauccaacggauagUcugaaa..............   1     1    Mm2
......................................................................ugugauccaacggauaggcugaaU..............   469   1    Mm2
......................................................................ugugauccaacggauaggcugaaag.............   11    0    Mm2
......................................................................ugugauccaacggauaggcugaaaA.............   47    1    Mm2
......................................................................ugugauccaacggauaggcugaaaU.............   95    1    Mm2
......................................................................ugugauccaacggauaggcugaGag.............   1     1    Mm2
......................................................................ugugauccaacggauaggcugaaGg.............   1     1    Mm2
......................................................................ugugauccaacggauaggcugaaaC.............   2     1    Mm2
......................................................................ugugauccaacggauaggcugaaagU............   21    1    Mm2
......................................................................ugugauccaacggauaggcugaaagA............   1     1    Mm2
......................................................................ugugauccaacggauaggcugaaagAa...........   1     1    Mm2
......................................................................ugugauccaacggauaggcugaaagGa...........   1     1    Mm2
.......................................................................gugauccaacggauaggc...................   1     0    Mm2
.......................................................................gugauccaacggauaggcu..................   1     0    Mm2
.......................................................................gugauccaacggauaggcug.................   7     0    Mm2
.......................................................................gugauccaacggauaggcugG................   1     1    Mm2
.......................................................................gugauccaacggauaggcugU................   14    1    Mm2
.......................................................................gugauccaCcggauaggcuga................   1     1    Mm2
.......................................................................gugauccaacggauaggUuga................   1     1    Mm2
.......................................................................gugauccaacggauaggcuga................   45    0    Mm2
.......................................................................Uugauccaacggauaggcuga................   1     1    Mm2
.......................................................................gugauccaacggauaggcugaU...............   1     1    Mm2
........................................................................ugauccaacggauaggcug.................   1     0    Mm2
........................................................................ugauccaacggauGggcuga................   1     1    Mm2
........................................................................ugauccaacggauaggcuga................   7     0    Mm2
........................................................................ugauccaacggauaggcugU................   8     1    Mm2
.........................................................................gauccaacggauaggcug.................   2     0    Mm2
.........................................................................gauccaacggauaggcugU................   1     1    Mm2
.........................................................................Uauccaacggauaggcuga................   1     1    Mm2
..........................................................................auccaacggauaggcugaaagc............   1     0    Mm2

...................................agcccaauccauguggac.......................................................   11    0    Mm1
...................................agcccaauccauguggaccaua...................................................   7     0    Mm1
...................................agcccaauccauguggaccauau..................................................   8     0    Mm1
...................................agcccaauccauguggCccauau..................................................   1     1    Mm1
....................................gcccaauccauguggaccauaug.................................................   2     0    Mm1
.....................................................................Cugugauccaacggauaggcugaa...............   9     1    Mm1
......................................................................ugugauccaaUggauagg....................   1     1    Mm1
......................................................................ugugauccaacggauagA....................   49    1    Mm1
......................................................................ugugauccaacggauagg....................   154   0    Mm1
......................................................................ugugauccaacggauagU....................   118   1    Mm1
......................................................................ugugauccaUcggauagg....................   1     1    Mm1
......................................................................ugugauccaacggauagCc...................   1     1    Mm1
......................................................................ugugauccUacggauaggc...................   1     1    Mm1
......................................................................ugugauccaacggauaggU...................   28    1    Mm1
......................................................................ugugauccaacggauaUgc...................   1     1    Mm1
......................................................................ugugauccaacggauagUc...................   3     1    Mm1
......................................................................ugugauccaacggauaggc...................   335   0    Mm1
......................................................................ugugauccaacggauagAc...................   3     1    Mm1
......................................................................ugugauccaaGggauaggc...................   1     1    Mm1
......................................................................ugugauccaacggauaAgc...................   1     1    Mm1
......................................................................ugugauccaacggauaCgc...................   1     1    Mm1
......................................................................ugugaCccaacggauaggcu..................   2     1    Mm1
......................................................................Ngugauccaacggauaggcu..................   3     1    Mm1
......................................................................ugugauccGacggauaggcu..................   1     1    Mm1
......................................................................ugugauccaacggauagAcu..................   21    1    Mm1
......................................................................ugugauccaaAggauaggcu..................   4     1    Mm1
......................................................................ugugauccaacggauagCcu..................   20    1    Mm1
......................................................................ugugauNcaacggauaggcu..................   1     1    Mm1
......................................................................ugugauccaacggaGaggcu..................   1     1    Mm1
......................................................................uAugauccaacggauaggcu..................   2     1    Mm1
......................................................................ugugauccaacggauagUcu..................   20    1    Mm1
......................................................................ugugauccaacggauaggcA..................   14    1    Mm1
......................................................................ugugauccaacggauaggcC..................   1     1    Mm1
......................................................................ugugauccUacggauaggcu..................   2     1    Mm1
......................................................................Ggugauccaacggauaggcu..................   1     1    Mm1
......................................................................ugugaNccaacggauaggcu..................   1     1    Mm1
......................................................................ugugauccaacggaAaggcu..................   1     1    Mm1
```

```
                                   Star                              Mature
ggucauggaaucacgccuagaccuguuuuccuuucagcccaauccauguggaccauaugucagaaaaucaugugauccaacggauaggcugaaagcaaaacaaaaugc
......................................................................ugugauccaacggauaggcG..................  24     1   Mm1
......................................................................ugugauccaaUggauaggcu..................  4      1   Mm1
......................................................................Agugauccaacggauaggcu..................  1      1   Mm1
......................................................................ugugauccaacggauaCgcu..................  16     1   Mm1
......................................................................ugCgauccaacggauaggcu..................  1      1   Mm1
......................................................................ugugauccaacggauaggcu..................  4162   0   Mm1
......................................................................ugugauccaacggauaUgcu..................  5      1   Mm1
......................................................................ugugauccaGcggauaggcu..................  2      1   Mm1
......................................................................ugugauccaacggauGggcu..................  102    1   Mm1
......................................................................ugugauccaacggUuaggcu..................  1      1   Mm1
......................................................................ugGgauccaacggauaggcu..................  1      1   Mm1
......................................................................ugugauccaaGggauaggcu..................  3      1   Mm1
......................................................................ugugauccaacggauaAgcu..................  10     1   Mm1
......................................................................ugugauccaacggauaCgcug.................  6      1   Mm1
......................................................................ugugauccaacggauaggcug.................  2543   0   Mm1
......................................................................ugugauccaaUggauaggcug.................  2      1   Mm1
......................................................................ugugauccUacggauaggcug.................  4      1   Mm1
......................................................................Ngugauccaacggauaggcug.................  1      1   Mm1
......................................................................ugugauccaacggauaAgcug.................  4      1   Mm1
......................................................................ugugauccaacggauaggcAg.................  1      1   Mm1
......................................................................ugugauccaacUgauaggcug.................  1      1   Mm1
......................................................................ugugauccaacggGuaggcug.................  1      1   Mm1
......................................................................ugugauccaacggauaggcuC.................  11     1   Mm1
......................................................................ugugauccaacggauagAcug.................  17     1   Mm1
......................................................................ugugaucUaacggauaggcug.................  1      1   Mm1
......................................................................ugugauccaacggauaggcuU.................  39     1   Mm1
......................................................................ugugauccaacggauaggcuA.................  37     1   Mm1
......................................................................ugugauccaacgAauaggcug.................  1      1   Mm1
......................................................................Agugauccaacggauaggcug.................  1      1   Mm1
......................................................................ugugauccaacggauagUcug.................  12     1   Mm1
......................................................................ugugauGcaacggauaggcug.................  1      1   Mm1
......................................................................ugugauUcaacggauaggcug.................  1      1   Mm1
......................................................................ugGgauccaacggauaggcug.................  2      1   Mm1
......................................................................Cgugauccaacggauaggcug.................  2      1   Mm1
......................................................................ugugauccaacCgauaggcug.................  1      1   Mm1
......................................................................ugugauccaacggauagCcug.................  12     1   Mm1
......................................................................ugugauccaacggauaUgcug.................  6      1   Mm1
......................................................................ugugauccaaAggauaggcug.................  1      1   Mm1
......................................................................ugugauccaacgCauaggcug.................  1      1   Mm1
......................................................................ugugauccaacggauaggcGg.................  4      1   Mm1
......................................................................ugugauccaaGggauaggcug.................  4      1   Mm1
......................................................................ugugauccaacggauGggcug.................  80     1   Mm1
......................................................................ugugauccCacggauaggcug.................  1      1   Mm1
......................................................................ugAgauccaacggauaggcuga................  10     1   Mm1
......................................................................ugugauccaacggauaAgcuga................  94     1   Mm1
......................................................................ugugaucUaacggauaggcuga................  16     1   Mm1
......................................................................ugCgauccaacggauaggcuga................  3      1   Mm1
......................................................................ugugauccaacggGuaggcuga................  1      1   Mm1
......................................................................ugugauccaacUgauaggcuga................  7      1   Mm1
......................................................................ugugauccaacggaGaggcuga................  1      1   Mm1
......................................................................ugugauccaaGggauaggcuga................  51     1   Mm1
......................................................................ugugaCccaacggauaggcuga................  4      1   Mm1
......................................................................ugugauccaacggaCaggcuga................  3      1   Mm1
......................................................................ugugauAcaacggauaggcuga................  3      1   Mm1
......................................................................ugugauccaaAggauaggcuga................  65     1   Mm1
......................................................................ugugauccaacggauaCgcuga................  93     1   Mm1
......................................................................ugugauccaacggUuaggcuga................  2      1   Mm1
......................................................................ugugauccaacggCuaggcuga................  1      1   Mm1
......................................................................ugugauccaacCgauaggcuga................  1      1   Mm1
......................................................................ugugauccaacgCauaggcuga................  2      1   Mm1
......................................................................uguAauccaacggauaggcuga................  7      1   Mm1
......................................................................ugugauccaacggauagCcuga................  191    1   Mm1
......................................................................ugugauUcaacggauaggcuga................  6      1   Mm1
......................................................................uUugauccaacggauaggcuga................  5      1   Mm1
......................................................................ugugaAccaacggauaggcuga................  4      1   Mm1
......................................................................uguUauccaacggauaggcuga................  3      1   Mm1
......................................................................ugugauccaacgUauaggcuga................  5      1   Mm1
......................................................................ugugauccaacggauaggcuga................  37211  0   Mm1
......................................................................ugugauccaacggauaggAuga................  3      1   Mm1
......................................................................ugugauccaacggauaggcAga................  3      1   Mm1
```

```
                                   Star                               Mature
ggucauggaaucacgccuagaccuguuuuccuuucagcccaauccauguggaccauaugucagaaaaucaugugauccaacggauaggcugaaagcaaaacaaaaugc
......................................................................ugugauccaaUggauaggcuga................    44      1    Mm1
......................................................................ugugaGccaacggauaggcuga................    1       1    Mm1
......................................................................ugugauccaacggauaggGuga................    1       1    Mm1
......................................................................ugugUuccaacggauaggcuga................    3       1    Mm1
......................................................................Cgugauccaacggauaggcuga................    5       1    Mm1
......................................................................ugugauccaacggauGggcuga................    1009    1    Mm1
......................................................................ugugauccaacggauaggcugG................    156     1    Mm1
......................................................................Ngugauccaacggauaggcuga................    20      1    Mm1
......................................................................ugugCuccaacggauaggcuga................    9       1    Mm1
......................................................................ugugauccaGcggauaggcuga................    7       1    Mm1
......................................................................ugugauccaacgAauaggcuga................    1       1    Mm1
......................................................................ugugauccaacggauaggUuga................    8       1    Mm1
......................................................................ugugauccaacggauagUcuga................    179     1    Mm1
......................................................................ugugauccaacggauaggcugC................    25      1    Mm1
......................................................................ugugauccaacggauaggcCga................    8       1    Mm1
......................................................................uguCauccaacggauaggcuga................    1       1    Mm1
......................................................................ugugGuccaacggauaggcuga................    14      1    Mm1
......................................................................uAugauccaacggauaggcuga................    14      1    Mm1
......................................................................ugugauccaUcggauaggcuga................    3       1    Mm1
......................................................................Agugauccaacggauaggcuga................    3       1    Mm1
......................................................................ugGgauccaacggauaggcuga................    9       1    Mm1
......................................................................ugugauccaacAgauaggcuga................    9       1    Mm1
......................................................................ugugaucGaacggauaggcuga................    1       1    Mm1
......................................................................ugugauccaacggauaggcugU................    7383    1    Mm1
......................................................................ugugauccaacggauaggcuUa................    6       1    Mm1
......................................................................ugugauccaacggauaggcGga................    1       1    Mm1
......................................................................ugugaNccaacggauaggcuga................    4       1    Mm1
......................................................................ugugauccaacggauaUgcuga................    64      1    Mm1
......................................................................ugugauccCacggauaggcuga................    3       1    Mm1
......................................................................ugugauccaacggauaggcuCa................    3       1    Mm1
......................................................................ugugauNcaacggauaggcuga................    4       1    Mm1
......................................................................ugugauccaacggauUggcuga................    4       1    Mm1
......................................................................uCugauccaacggauaggcuga................    3       1    Mm1
......................................................................ugugauccaacggauaggcuAa................    4       1    Mm1
......................................................................ugugaucAaacggauaggcuga................    2       1    Mm1
......................................................................ugugauccGacggauaggcuga................    5       1    Mm1
......................................................................ugugauccaacggauagAcuga................    209     1    Mm1
......................................................................ugugauccUacggauaggcuga................    20      1    Mm1
......................................................................ugugauccaCcggauaggcuga................    4       1    Mm1
......................................................................ugugauGcaacggauaggcuga................    2       1    Mm1
......................................................................Ggugauccaacggauaggcugaa...............    1       1    Mm1
......................................................................ugGgauccaacggauaggcugaa...............    1       1    Mm1
......................................................................ugugauccaacggauagCcugaa...............    11      1    Mm1
......................................................................ugugaucUaacggauaggcugaa...............    2       1    Mm1
......................................................................uAugauccaacggauaggcugaa...............    1       1    Mm1
......................................................................ugugauccaUcggauaggcugaa...............    2       1    Mm1
......................................................................ugugauccaacggauaggcugaG...............    25      1    Mm1
......................................................................ugugauccaacggauaggcugaU...............    625     1    Mm1
......................................................................Agugauccaacggauaggcugaa...............    2       1    Mm1
......................................................................ugugauccaacggauaUgcugaa...............    8       1    Mm1
......................................................................ugugauccaaUggauaggcugaa...............    2       1    Mm1
......................................................................ugugauccaacggauagUcugaa...............    15      1    Mm1
......................................................................ugugauccaacggauaAgcugaa...............    8       1    Mm1
......................................................................ugugauccaacggauaCgcugaa...............    12      1    Mm1
......................................................................ugugauccaaGggauaggcugaa...............    8       1    Mm1
......................................................................uCugauccaacggauaggcugaa...............    1       1    Mm1
......................................................................uguAauccaacggauaggcugaa...............    2       1    Mm1
......................................................................ugugauccaacggauaggcugaa...............    2858    0    Mm1
......................................................................ugugauccaacggauagAcugaa...............    18      1    Mm1
......................................................................ugugauccaacggauGggcugaa...............    25      1    Mm1
......................................................................uUugauccaacggauaggcugaa...............    1       1    Mm1
......................................................................ugugauccaacggauaggcCgaa...............    1       1    Mm1
......................................................................ugugCuccaacggauaggcugaa...............    1       1    Mm1
......................................................................ugugauccaacggauaggcAgaa...............    1       1    Mm1
......................................................................ugugaucGaacggauaggcugaa...............    1       1    Mm1
......................................................................ugugauccaacggauaggcugUa...............    54      1    Mm1
......................................................................ugugauccaacggauaggcugaGa..............    1       1    Mm1
......................................................................ugugauccaacggauaggcugaaG..............    6       1    Mm1
......................................................................ugugauccaaUggauaggcugaaa..............    1       1    Mm1
......................................................................ugugauccaacggauaggcugaaC..............    1       1    Mm1
```

```
                                   Star                               Mature
ggucauggaaucacgccuagaccuguuuuccuuucagcccaauccauguggaccauaugucagaaaaucaugugauccaacggauaggcugaaagcaaaacaaaaugc
......................................................................uAugauccaacggauaggcugaaa..............   1     1    Mm1
......................................................................ugugauccaacggauaggcugaUa..............   12    1    Mm1
......................................................................ugugauccaacggauagAcugaaa..............   1     1    Mm1
......................................................................ugugauccaacggauaCgcugaaa..............   2     1    Mm1
......................................................................ugugauccaacggauaggcugaaU..............   166   1    Mm1
......................................................................ugugauccaacggauaggcugaaa..............   173   0    Mm1
.......................................................................gugauccaacggauaggcugU................   3     1    Mm1
.........................................................................gauccaacggauaggcug.................   9     0    Mm1
```
